# Detailed Experimental and In Silico Investigation of Indomethacin Binding with Human Serum Albumin Considering Primary and Secondary Binding Sites

**DOI:** 10.3390/molecules28072979

**Published:** 2023-03-27

**Authors:** Mohd Sajid Ali, Jayaraman Muthukumaran, Monika Jain, Mohammad Tariq, Hamad A. Al-Lohedan, Abdullah Saad S. Al-Sanea

**Affiliations:** 1 Department of Chemistry, College of Science, King Saud University, P.O. Box 2455, Riyadh 11451, Saudi Arabia; 2 Department of Biotechnology, School of Engineering and Technology, Sharda University, Greater Noida 201310, India; 3 LAQV-REQUIMTE, Departamento de Química, Faculdade de Ciências e Tecnologia, Universidade NOVA de Lisboa, 2829-516 Caparica, Portugal

**Keywords:** albumin, indomethacin, primary binding site, secondary binding site, fluorescence, molecular dynamics

## Abstract

The interaction of indomethacin with human serum albumin (HSA) has been studied here considering the primary and secondary binding sites. The Stern–Volmer plots were linear in the lower concentration range of indomethacin while a downward curvature was observed in the higher concentration range, suggesting the presence of more than one binding site for indomethacin inside HSA due to which the microenvironment of the fluorophore changed slightly and some of its fraction was not accessible to the quencher. The Stern–Volmer quenching constants (K_SV_) for the primary and secondary sites were calculated from the two linear portions of the Stern–Volmer plots. There was around a two-fold decrease in the quenching constants for the low-affinity site as compared to the primary binding site. The interaction takes place via a static quenching mechanism and the K_SV_ decreases at both primary and secondary sites upon increasing the temperature. The binding constants were also evaluated, which show strong binding at the primary site and fair binding at the secondary site. The binding was thermodynamically favorable with the liberation of heat and the ordering of the system. In principle, hydrogen bonding and Van der Waals forces were involved in the binding at the primary site while the low-affinity site interacted through hydrophobic forces only. The competitive binding was also evaluated using warfarin, ibuprofen, hemin, and a warfarin + hemin combination as site markers. The binding profile remained unchanged in the presence of ibuprofen, whereas it decreased in the presence of both warfarin and hemin with a straight line in the Stern–Volmer plots. The reduction in the binding was at a maximum when both warfarin and hemin were present simultaneously with the downward curvature in the Stern–Volmer plots at higher concentrations of indomethacin. The secondary structure of HSA also changes slightly in the presence of higher concentrations of indomethacin. Molecular dynamics simulations were performed at the primary and secondary binding sites of HSA which are drug site 1 (located in the subdomain IIA of the protein) and the hemin binding site (located in subdomain IB), respectively. From the results obtained from molecular docking and MD simulation, the indomethacin molecule showed more binding affinity towards drug site 1 followed by the other two sites.

## 1. Introduction

The uses of nonsteroidal anti-inflammatory drugs in various diseases such as pain, inflammation, fever, various types of cancers, cardiovascular diseases, and problems associated with peripheral and central nervous systems are well known [1]. Indomethacin is an important FDA-approved drug used to cure fever, pain, stiffness, and swelling from inflammation. It is effective in moderate to severe pain relief [2]. Indomethacin can either be given to patients orally in the form of tablets or suspensions, via intravenous infusion, or via rectal suppository [3].

The intravenous infusion of the drugs in the body releases them into the bloodstream directly, which may lead to their binding and interactions with blood components. Blood contains a lot of proteins that are responsible for the binding of various substances and carrying out various therapeutic functions of which serum albumin is the principal component of the blood proteins [4].

**Figure 1:**
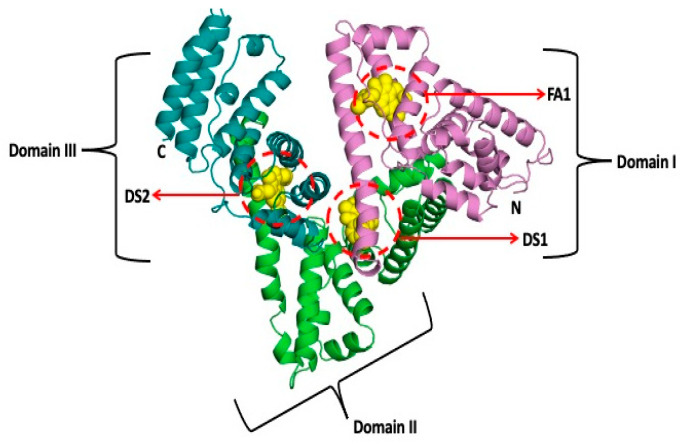
Three-dimensional structure of human serum albumin with drug and fatty acid binding sites.

Human serum albumin (HSA) is a large globular protein that has a molecular mass of 67 kDa and consists of 585 amino acid residues. It possesses the unusual capability of ligand binding and works as a transporter of several endogenous and exogeneous compounds such as bilirubin, fatty acids, and a large number of drug substances [5]. As stated by Fanali and coworkers, “HSA is widely used clinically to treat several diseases, including hypovolemia, shock, burns, surgical blood loss, trauma, hemorrhage, cardiopulmonary bypass, acute respiratory distress syndrome, hemodialysis, acute liver failure, chronic liver disease, nutrition support, resuscitation, and hypoalbuminemia” [6]. The three-dimensional structure of HSA with drug binding (DS1 and DS2) and fatty acid binding (FA1) sites are given in Figure 1.

HSA and its BSA counterpart are among the most studied protein and its binding with various types of substances is well known [7,8,9,10,11,12,13,14,15,16,17,18,19,20,21,22,23,24,25,26]. Understanding the binding mechanism of a drug is necessary to understand its pharmacokinetics and pharmacodynamics. Albumin binds with several ligands at different extents and the binding level of a compound may vary from very strong to weak depending on the various affinities responsible for the interaction [7,8,9,10,11,12,13,14,15,16,17,18,19,20].

In this work, we have studied the binding of HSA with the therapeutically important drug indomethacin. The interaction of these has been the topic of concern at various instances and was appraised using several methods [27,28,29,30,31,32,33,34,35,36,37]. However, the important aspect of the inner filter effect (IFE) correction was ignored in the studies, which involved methods such as fluorescence and/or UV absorption, to name a few [34,35,36,37]. In our recent studies, we have reported that the IFE, largely, affects the fluorescence quenching data and its level is dependent on the values of absorbances at the excitation and emission wavelengths [7,14,15,38]. Thus, we have reassessed the binding of HSA with indomethacin using difference UV absorption and intrinsic fluorescence spectroscopies. It was also reported that indomethacin has two binding sites inside HSA [39]: (i) a primary binding site, which is the one considered as drug site 1 (DS1) and (ii) a secondary binding site (FA1), which are given in Figure 1. Therefore, it would be interesting to see the competitive binding of indomethacin in the presence of the site markers of these sites when the later is present alone or in combination with each other. Thus, we have seen the binding of indomethacin with HSA complexed with warfarin (DS1 marker), hemin (FA1 marker), and warfarin + hemin. Although, the competitive binding using ibuprofen as a DS2 marker was also seen just for the sake of knowing whether indomethacin has any preferential binding at DS2 or not. In the literature, there are enormous studies in which the competitive binding assays of HSA have been studied using a single site marker; however, to the best of our knowledge, no competitive study has been performed using two site markers of the drug, which has two binding sites inside HSA. Moreover, the prevailing interactions have also been screened using computational methods, which are very popular nowadays to study the binding of biomolecules and small ligands. For the computational studies, we have employed the basic computational approach—molecular docking, as well as the advanced—molecular dynamics simulations. This study will be an advancement of the pioneering works previously reported by various authors and research groups on the albumin–indomethacin binding [27,28,29,30,31,32,33,34,35,36,37].

## 2. Results and Discussions

### 2.1. Experimental Investigation of Indomethacin Binding with HSA

**Figure 2:**
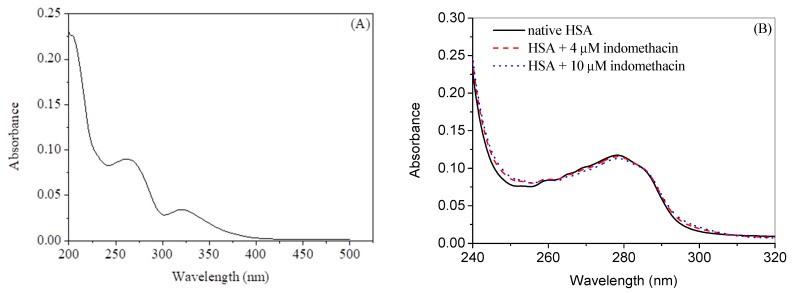
(**A**) UV absorption spectrum of 5 µM indomethacin in 20 mM buffer of pH 7.4. (**B**) Difference UV absorption spectra of HSA in absence and presence of indomethacin at 25 °C.

The UV absorption spectrum of pure indomethacin in 20 mM tris-HCl buffer of pH 7.4 is given in Figure 2A, which shows two peaks at around 260 nm and 320 nm [40]. Changes in the UV absorption spectrum can be utilized to understand the interaction between the biomolecule and ligand [41]. Usually, difference spectra (in which the contribution of the ligand absorbance is subtracted from the collective absorbance of the complex) give more straightforward ideas as compared to normal spectra [15,16,38,42,43,44] and such difference UV absorption spectra of HSA in absence and presence of indomethacin in the wavelength range of 240 nm to 320 nm are given in Figure 2B and the corresponding observed spectra (without subtracting the indomethacin contribution) are given in Figure S1, supplementary materials. The observed spectra show a large hyperchromic shift owing to the significant absorption of indomethacin alone in the scrutinized wavelength range; however, the unerring change in the UV absorption profile was obtained (Figure 2B) in case of difference spectra, which confirms the complex formation between the protein and drug.

**Figure 3:**
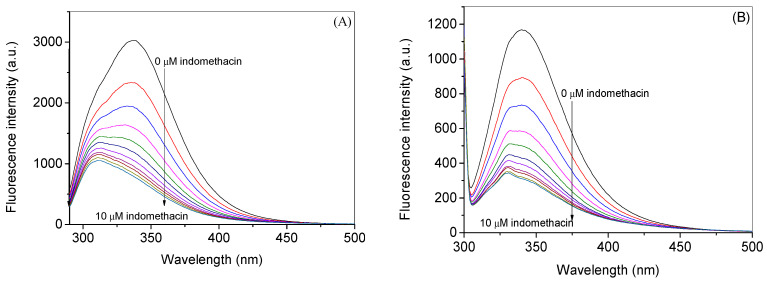
Observed fluorescence spectra of HSA in the absence and presence of indomethacin at λ_ex_ of 280 nm (**A**) and 295 nm (**B**) at 25 °C. [HSA] = 3 µM and [indomethacin] = 0 (black) 1.0, 2.0, 3.0, 4.0, 5.0, 6.0, 7.0, 8.0, 9.0, and 10.0 µM.

**Figure 4:**
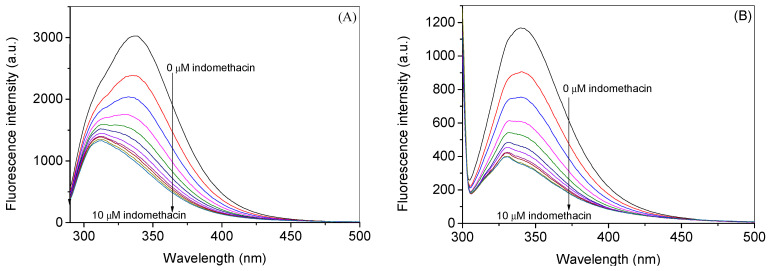
Corrected fluorescence spectra of HSA in the absence and presence of indomethacin at λ_ex_ of 280 nm (**A**) and 295 nm (**B**) at 25 °C. [HSA] = 3 µM and [indomethacin] = 0. 1.0, 2.0, 3.0, 4.0, 5.0, 6.0, 7.0, 8.0, 9.0, and 10.0 µM.

**Figure 5:**
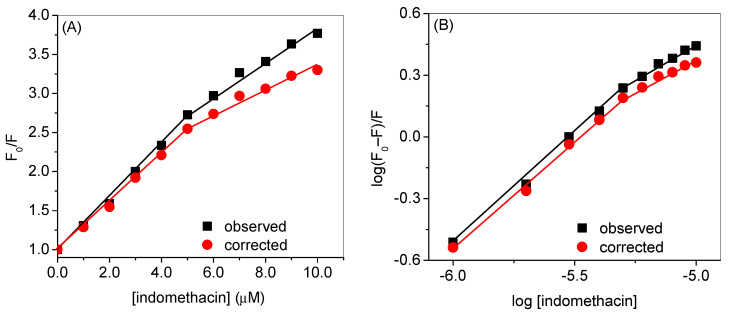
(**A**) Stern–Volmer plots of HSA–indomethacin interaction and (**B**) plots of log (F_0_–F)/F versus log [indomethacin] at the excitation wavelength of 295 nm for the observed and corrected data.

**Figure 6:**
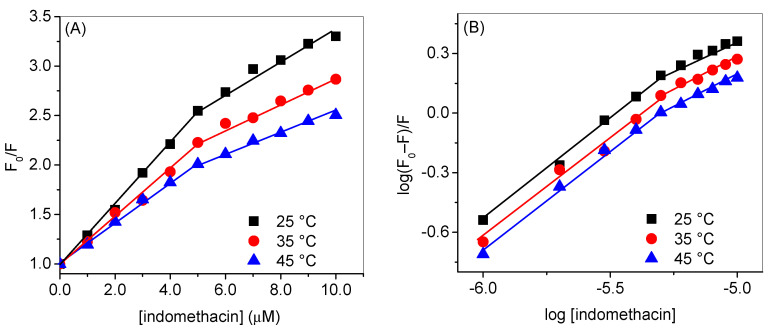
(**A**) Stern–Volmer plots of HSA-indomethacin interaction and (**B**) Plots of log (F_0_–F)/F versus log [indomethacin] at the excitation wavelength of 295 nm at various temperatures.

HSA has an intrinsic fluorescence property due to the presence of fluorescence residues such as tryptophan and tyrosine, which emit at around 340 nm and 315 nm, respectively, when excited at 280 nm; however, the latter shows minimal fluorescence as compared to the former [45]. Tryptophan alone can be excited at 295 nm to yield the maximum emission at 340 nm. When any molecule is added to the protein solution, the fluorescence property of the fluorophore present in the latter may change due to the changes in its microenvironment that depend on the interaction property of the added molecule [46]. In general, the decrement or quenching of the fluorescence takes place due to the energy transfer from the fluorophore to the small molecule (quencher) [47]. Figure 3A,B displays the observed fluorescence profiles of HSA at 25 °C with various concentrations of indomethacin at 280 nm and 295 nm excitation wavelengths, respectively, whereas their corresponding corrected spectra (using Equation S1) are shown in Figure 4A,B. Since there was almost no change in the relative fluorescence intensities of HSA (Figures S2 and S3) at two excitation wavelengths, 295 nm was selected as the excitation wavelength for further analyses. Furthermore, the observed fluorescence spectra at 35 °C and 45 °C at the excitation wavelength of 295 nm are given in Figures S4 and S5, whereas the corrected ones are given in Figures S6 and S7. The analyses of the fluorescence quenching data have been performed in two ways: (i) in the first set of experiments, we have shown the difference between the values of various constants obtained from the observed and corrected data, and their figures are displayed in Figure 5 and the data are given in Table 1; (ii) in the second set, we have shown the effect of temperature on the same parameters (Figure 6 and Table 2).

Quenching in the fluorescence emission intensity can be quantified by means of the well-known Stern–Volmer equation, which can be given as [48]:

(1) $$\frac{F_{0}}{F}=1+K_{SV}[Q]=1+K_{q}\tau_{0}[Q]$$

(2) $$Kq=\frac{K_{SV}}{\tau_{0}}$$

where *F_0_,* and *F* are the fluorescence intensities of HSA in the absence and presence of quencher (indomethacin), [*Q*] the concentration of quencher, and *K_SV_*, *K_q_*_,_ and *τ_0_* are the Stern–Volmer quenching constant, the biomolecular quenching constant, and the lifetime of the fluorophore in the absence of quencher (in this case 5.9 × 10^−9^ s^−1^ according to [16]), respectively. From the linear regression of Equation (1), i.e., the plot of *F*_0_/*F* vs. [Q], the values of *K_SV_* can be calculated.

The Stern–Volmer plots of HSA quenching by indomethacin at the excitation wavelength of 295 nm are plotted in Figure 5A for both observed and corrected data. These curves show negative deviation from linearity, mainly in the higher concentration range of indomethacin, and concave towards the x-axis. This phenomenon usually happens when there is more than one tryptophan residue with a distinct environment and different accessibility to the quencher [45,49,50], but it is well known that HSA has only one tryptophan residue located in the subdomain IIA, thus, there is a possibility of the existence of more than one binding site for indomethacin inside HSA [51], and the larger distance of secondary binding site in comparison to the primary one reduced the quenching efficiency, thus, a negative deviation in the Stern–Volmer plots could be expected [52,53,54]. Silva and coworkers have suggested that the binding of a ligand to the HSA may lead to the possible conformational change followed by the exposition of lower affinity binding sites [55]. HSA is known to possess several binding sites associated with the binding of fatty acids [39]; however, in principle, there are two drug binding sites designated as drug site 1, located in subdomain IIA, and drug site 2, situated in subdomain IIIA [56]. It has also been reported earlier through crystallographic studies of HSA in complexation with various drugs that indomethacin primarily binds in drug site 1 while it also has a secondary site which is located in subdomain IB and designated as the fatty acid binding site 1 or hemin binding site [39]. There are several crystal structures of HSA with various ligands that were reported previously [39], particularly the PDB IDs of 2BXM and 2BXK, which are related to the HSA complexed with indomethacin. Thus, from the pattern observed in the Stern–Volmer plots of the HSA–indomethacin interaction, the presence of two binding sites (primary and secondary) can be proposed for indomethacin within HSA and we have evaluated two quenching constants for each set of quenching experiments considering the primary and secondary sites from the first and second linear portions of the curves, respectively (Figure 5A), where the curves have been plotted for observed and corrected data at 25 °C, and Figure 6A in which the plots are given for the corrected data at various temperatures) [57]. There was, however, not too much but a fair difference between the values of observed and corrected constants (Table 1). As we have discussed, we have evaluated two K_SV_s from the two linear portions of the Stern–Volmer plots and we have designated them K_SV1_ (for the primary binding site) and K_SV2_ (for the secondary binding site) [57], and the values of the former were around two-fold higher than that of the latter. While discoursing the type of quenching, it is readily apparent from the values of K_q_s (given in Table S1) that they are much higher than the diffusion-controlled limit 1 × 10^10^ mol^ࢤ1^ s^ࢤ1^ and the converse dependency of both K_SV_s on the temperature that the mechanism involves in the interaction is the static type.

The binding constants (*k_b_*) and the number of binding sites (*n*) could be evaluated using the double logarithmic equations which are given as [58]:

(3) $$log\frac{F_{0}-F}{F}=logk_{b}+nlog[Q]$$

The plots of *log*(*F*_0_–*F*)/*F* vs. log[indomethacin] are given in Figure 5b (for observed and corrected data at 25 °C) and 5 (B) (for corrected data at various temperatures) and the respective values of *k_b_* and n are enlisted in Table 1 and Table 2. In this case too, we have observed the downward curvature, which can be parted in two straight lines corresponding to the two different sites. The binding constants of the primary site were very high compared to the binding constants of the secondary site.

**Figure 7:**
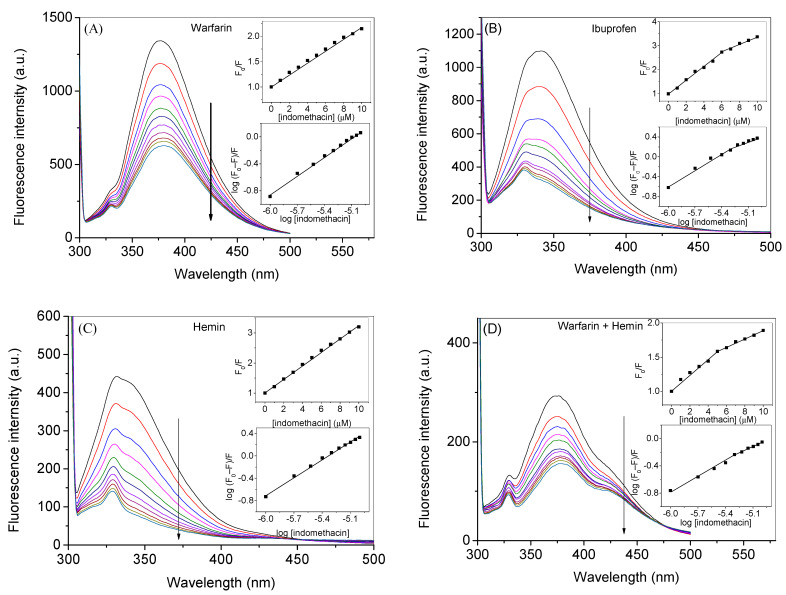
Corrected fluorescence spectra of HSA–warfarin (**A**), HSA–ibuprofen (**B**), HSA–hemin (**C**), and HSA–warfarin–hemin (**D**), systems in absence and presence of indomethacin at λ_ex_ of 295 nm at 25 °C. [HSA] = 3 µM and [indomethacin] = 0. 1.0, 2.0, 3.0, 4.0, 5.0, 6.0, 7.0, 8.0, 9.0, and 10.0 µM.

To gain more insight into the binding sites of indomethacin inside HSA, we have performed competitive binding experiments using the site markers of drug site 1 (warfarin), drug site 2 (ibuprofen), and fatty acid binding site 1 (hemin). Additionally, we have also used a combination of warfarin and hemin to see the combined effect of both (when primary as well as secondary sites are already occupied) on the binding of indomethacin with HSA (Figure 7, only corrected spectra are given in the main text; observed spectra are given in the supporting information (Figures S8–S11)). Surprisingly, there was a straight line in the case of the binding of indomethacin with the HSA–warfarin ternary as well as with hemin–HSA ternary complexes, while in the case of binding with HSA–ibuprofen ternary and HSA–warfarin-hemin quaternary complexes, there was a downward curvature in the plot. The corresponding quenching and binding constant plots are given in the insets of all figures associated with the site markers in Figure 7. The presence of straight lines in the case of the HSA–warfarin ternary as well as with the hemin–HSA ternary systems can be understood on the basis that in both cases one corresponding site was already occupied by the site marker and only one binding site was free for indomethacin. However, the binding of indomethacin with HSA was weaker in the presence of warfarin as compared to the presence of hemin, which suggests that the binding affinity of indomethacin was higher with site 1 in comparison to the hemin site (Table 3). It was also observed that the binding constant at the hemin binding site was higher in the presence of warfarin as compared to the one observed for free HSA. This can be explained on the basis that DS1 and the hemin binding site are allosterically coupled with each other [6] and the ligand present at one site may affect the binding of the ligand at the other site [59] due to the possible conformational changes after binding with one ligand. Thus, it can be supposed that the presence of warfarin increased the affinity of indomethacin at the hemin binding site. There was almost no change or a very small change in the quenching and binding constants when the binding of indomethacin was seen with the HSA–ibuprofen ternary complex. The quenching and binding constants were lowest in the case of the interaction of indomethacin with the HSA–warfarin–hemin system comprehensibly due to the occupancy of both primary and secondary binding sites; because of that, indomethacin confronted more competition for its binding inside HSA. The binding of indomethacin at various sites described in this section has also been studied using molecular docking and molecular dynamics simulations, which are discussed in the in silico section of the manuscript.

**Figure 8:**
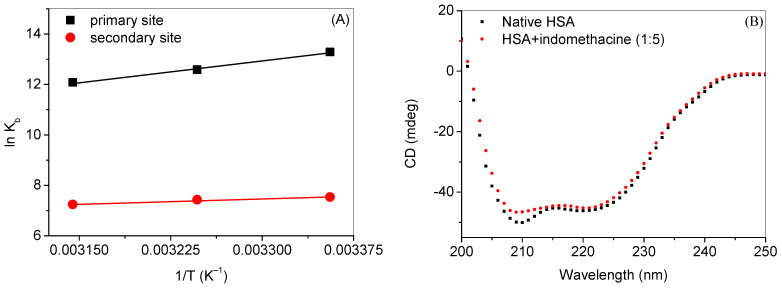
(**A**) Van’t Hoff plots of HSA–indomethacin interaction for primary and secondary sites. (**B**) Far-UV CD spectra of HSA (3 µM) in the absence and presence of indomethacin at 25 °C.

Thermodynamic parameters (as enthalpy change (Δ*H*), entropy (Δ*S*) and free energy change (Δ*G*)) were also evaluated using the well-known Van’t Hoff equations (given in Equations S2 and S3) and Van’t Hoff plots (Figure 8A) for the binding at primary as well as secondary sites (Table 4). The binding of indomethacin at the primary site was highly energetically feasible with large negative values of both enthalpy as well as entropy. However, Δ*G* and Δ*H* in the case of the binding at the secondary site were much less with a positive value of Δ*S.* From the values of thermodynamic parameters, it can be concluded that the interaction of HSA at the primary site is producing a more stable and less disordered complex, while the interaction at the secondary site is making a less stable and more disordered complex. The positive values of Δ*H* and Δ*S* are associated with the predominance of hydrophobic forces; however, the hydrogen bonding predominates when the values of these parameters are negative. In the case of DS1, both values are found to be negative, which means that the hydrogen bonding is the predominating force involved in the binding at this site. Although, there might be other forces involved, although less dominating [60]. In the case of the secondary binding site, it was revealed from the values of the thermodynamic parameters that both the hydrogen bonding as well as the hydrophobic forces played a major role in the interaction.

Secondary structural analysis was also performed using the far-UV CD method. It is very well known that HSA is an α-helical protein with around 67% α-helical contents [61]. The far-UV CD spectra of HSA in the absence and presence of indomethacin are given in Figure 8B. The far-UV CD spectrum of native HSA is a characteristic spectrum of an α-helical protein with two negative peaks at 208 nm and 222 nm. The addition of a small amount of indomethacin (1 and 2 µM) in HSA (data not shown) did not influence the secondary structure of the latter, although a higher concentration (5 µM) affects the secondary structure and induced partial unfolding of the protein.

### 2.2. In Silico Investigation of Indomethacin Binding with HSA

A molecular docking calculation was performed between indomethacin and three sites (drug site 1, drug site 2, and fatty acid binding site 1) of HSA. Further, the MD simulation is used to understand the structural stability of protein and protein–ligand complexes by analyzing various structural parameters, namely, the root mean square deviation (RMSD), root mean square fluctuation (RMSF), radius of gyration (Rg), intermolecular hydrogen bonds, solvent accessible surface area (SASA), and essential dynamics based on principal component analysis (PCA). Together, the results obtained from molecular docking and MD simulation, the details about the binding affinity, and the structural stability behavior of indomethacin towards three sites of HSA were studied at the molecular level. The estimated free energy of binding (Δ*G*) estimated inhibition constant (*Ki*) and estimated binding affinity of indomethacin towards HSA was given in Table 5.

From the results of three independent docking calculations, the indomethacin molecule showed more binding affinity (−10.1 kcal/mol) towards DS1 followed by FA1 (−8.4 kcal/mol) and DS2 (−7.96 kcal/mol). These results corroborated with residue interaction analysis, which is given in Table 6. Indomethacin made two intermolecular hydrogen bonds with tryptophan and arginine residues at the position of 214 and 218, whereas it made a single hydrogen bond with N391 residue in the case of DS2. This molecule did not form any hydrogen bonds with FA1; however, it forms several hydrophobic interactions with FA1. The results obtained from the computational studies are in good agreement with what is obtained from the experimental methods (Table 4) which, after assessing the values of ΔG, show that the binding of indomethacin with HSA at the DS1 was more favorable as compared to the binding at the hemin binding site (FA1). Here, in the case of the driving forces involved in the binding, we have observed a slight discrepancy between experimental and docking investigations, which might be due to the fact that in the case of the latter, only the ligand and receptor (rigid) is selected, whereas in the case of former, a lot of factors are present such as the solvent, buffer, flexible receptor, etc. [62,63].

**Figure 9:**
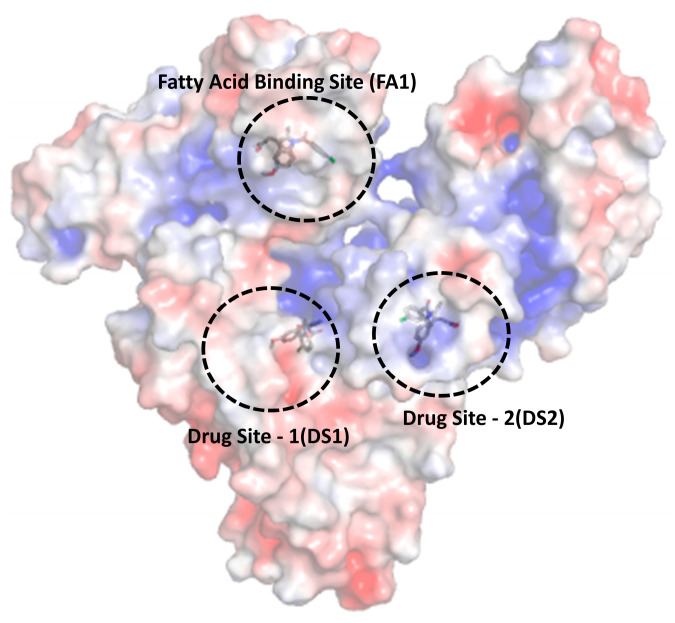
Electrostatic surface potential map of three sites of human serum albumin with indomethacin. Red (negative potential), blue (positive potential), and white (neutral potential).

Furthermore, the electrostatic surface potential map analysis of indomethacin-bound HSA complexes (Figure 9) using PyMOL explained the nature of the binding of the drug molecule towards the target protein. From this result, it has been observed that most of the portion of the indomethacin molecule was principally oriented in positively charged amino acid residues of HSA in the case of DS1, whereas it oriented in both positive and negative charged residues of HSA in the case of the other two sites, namely, DS2 and FA1.

**Figure 10:**
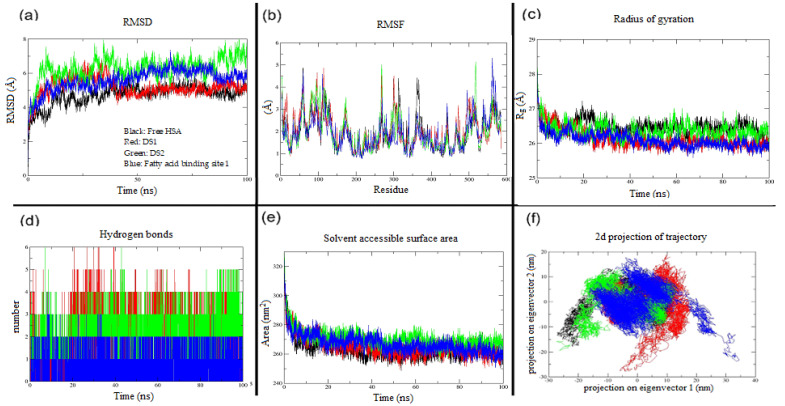
Molecular dynamics simulation results of free HSA and three sites of human serum albumin with indomethacin (**a**) root mean square deviation, (**b**) root mean square fluctuation, (**c**) radius of gyration, (**d**) intermolecular hydrogen bonds (**e**) solvent accessible surface area, and (**f**) essential motion using principal component analysis (color codes: black—free HSA, red—DS1, green—DS2, and blue—fatty acid binding site 1).

In order to validate the results obtained from three independent molecular docking calculations of HSA with indomethacin, we have subsequently processed these three complexes into MD simulation along with free HSA. The molecular docking calculation is a semi-flexible approach that considers protein as a rigid entity and ligand as a flexible entity. To some extent, a few residues of the protein are treated as flexible, but not the entire protein. This is one of the major limitations of molecular docking studies. To overcome this, we have additionally performed MD simulations for HSA along with indomethacin bound to three important sites of HSA to understand the ligand-induced structural stability, ligand-induced binding affinity, and ligand-induced conformational changes in the structure of HSA. The MD simulation results depicted that there is no significant alteration in the structural stability of HSA while binding indomethacin to DS2 and FA1. In contrast, the indomethacin-bound DS1 site of HSA showed a similar RMSD pattern as native protein from 30 ns to the end of the MD simulation period, which indicated that HSA maintains the overall structural stability upon the binding of indomethacin to DS1. Firstly, we have performed RMSD analysis of four states of HSA (a) free HSA, (b) indomethacin bound to DS1 of HSA, (c) indomethacin bound to DS2 of HSA, and (d) indomethacin bound to FA1 of HSA. This analysis is very important to measure the structural stability behavior of HSA and HSA–indomethacin complexes. In the RMSD graph of indomethacin bound to DS1 (Figure 10A), we have observed a stable and consistent pattern from 30 ns onwards and it maintains until the end of the simulation period as free HSA (4 to 4.5 Å). In contrast, indomethacin bound to the other two sites showed higher RMSD unto 7 Å. Overall, in comparison with all three sites, indomethacin showed a similar trend of the RMSD pattern towards DS1 as a native HSA. This result corroborates well with molecular docking calculations of the DS1 site of HSA with indomethacin. The average RMSD values of four states of MD simulation are given in Table 7.

The RMSF analysis (Figure 10B) is used to identify the flexible and non-flexible residues of HSA. Upon the binding of indomethacin to the DS1 site, HSA does not significantly increase or decrease the flexibility but maintains the same state as free HSA. However, slightly higher flexibility was recorded in the other two states (DS2 and FA1) of HSA. The average RMSF value of the four states of MD simulation is 1.9 Å. The top five fluctuating residues of the four states of HSA are as follows: free HSA (A59, E60, D301, A362, and K313), indomethacin-bound DS1 of HSA (E95, E97, R114, Q268, and D301), indomethacin-bound DS2 of HSA (4K, Q268, L516, S517, and E518) and indomethacin-bound FA1 of HSA (E60, N111, D562, D563, and E565). The radius of gyration (Figure 10C) analysis is mainly used to measure the shape, compactness, and folding properties of protein upon the influence of ligand binding. Interestingly, the average Rg (Table 7) values of indomethacin-bounded HSA structures are less in comparison with free HSA, which explained that the ligand molecule did not affect the compactness and folding properties of HSA.

Very interestingly, we have observed the maximum number of three intermolecular hydrogen bonds between HSA and indomethacin at binding site DS1 predicting significant binding affinity at DS1, (Figure 10D and Table 7). Moreover, we have presented an interaction analysis of MD-simulated structures of HSA bound with indomethacin in the supplementary data (Figures S14–S16). In order to understand the ligand-induced conformation changes, we have additionally performed two important analyses, namely, solvent accessible surface area (Figure 10E) and secondary structural composition (Table 8) of HSA. Both of these analyses suggested that the indomethacin molecules do not cause significant changes in the structure of HSA and it does not alter the structural stability and flexibility of HSA. Apart from the aforementioned global dynamics analysis, we have additionally performed essential dynamics of HSA and indomethacin-bound complexes based on principal component analysis (PCA) (Figure 10F). The PCA was performed to capture the biologically relevant dominant motions from the global trajectory of free and indomethacin-bound HSA systems. The PCA extracts the dominant motions by converting the higher dimensional global dynamics data into lower dimensional essential dynamics (ED) data. In the end, it lists eigenvectors ranked according to their contribution to the ED. This result explained that indomethacin-bound HSA complexes experienced greater essential dynamics than the unbound HSA. Combining the results on UV-CD and secondary structure analysis using MD simulation, it has been found that HSA is rich in alpha helices and upon binding of indomethacin to HSA, it does not cause any significant changes in terms of various secondary structural compositions. The in silico results corroborate with the experimental results. The time-averaged structural properties obtained from MD simulations of free HSA and indomethacin-bound HSA are presented in Table 7. Overall, the results explained that in comparison with DS2 and FA1, indomethacin prefers to bind to DS1 of HSA, and moreover, upon the binding of the drug molecule to DS1 of HSA, it maintains overall structural stability, folding properties, and compactness.

## 3. Materials and Methods

HSA essentially fatty acid free (≥99%, A3782) and indomethacin (≥99%, I7378) were purchased from Sigma-Aldrich Co., St. Louis, MO, USA. The studies were performed in the 20 mM tris-HCl buffer of pH 7.4. UV absorption spectra were collected on a Perkin-Elmer Lambda-45 double beam UV-visible spectrophotometer using quartz cells of 1 cm. The intrinsic fluorescence studies were performed on the Hitachi F-7000 fluorescence spectrometer. Far UV-CD spectra were screened by exploiting the Jasco J-815 CD spectrophotometer. The rest of the details about the experimental part are given in the supporting information.

The competitive binding assays were performed using fluorescence spectroscopy by selecting a similar instrument setting as described in the last paragraph. Before the measurements, HSA (3 µM) was mixed with the site marker (5 µM) and the sample was incubated for several hours to ensure complete binding. The site markers were taken in slight excess to make certain the complete occupancy of the particular binding site(s).

Molecular docking or in silico interaction studies are computational procedures, which were used to understand the binding orientation using Auto Dock Vina [64]. The binding mode, crucial intermolecular interactions, vital interacting residues, and estimated free energy of binding (Δ*G*), estimated inhibition constant (*Ki*) of a ligand molecule towards human serum albumin (HSA) were also computed by molecular docking studies. In order to understand all possible orientations of indomethacin towards three sites of BSA, we chose the ligand-free form of HSA for computational studies. In this regard, the three-dimensional structure of HSA was retrieved from Research Collaboratory Structural Bioinformatics—Protein Data Bank (RCSB—PDB) (www.rcsb.org; accessed on 10th September 2021) with the PDB ID of 1AO6. Then, the protein structure of HSA was subjected to a protein preparation step using Molecular Graphics Laboratory (MGL) tools (http://mgltools.scripps.edu/; accessed on 10 September 2021) [65], which includes the following steps: (a) adding polar hydrogens, (b) merging non-polar hydrogens, and (c) assigning each atom with Kollman partial charges and recorded into PDBQT (XYZ Coordinates + Partial charge + atom type) format. Subsequently, the three-dimensional structure of indomethacin (ID: DB00328) was retrieved from the DrugBank database (https://go.drugbank.com/drugs/DB00328; accessed on 10 September 2021) and subjected to ligand preparation steps, which include (a) the addition of polar hydrogens, (b) merging of non-polar hydrogens, and (c) addition of gasteiger charges, and finally being saved into PDBQT format using MGL tools. Once the protein and ligand preparation steps were over, the three receptor grid maps were generated based on (a) drug site 1 (Center_X: 30.00 Å, Center_Y: 12.17 Å and Center_Z: −2.82 Å, Size_X: 34.42 Å, Size_Y: 40.89, and Size_Z: 34.25 Å; exhaustiveness: 8; energy range: 3) (b) drug site 2 (Center_X: 8.96 Å, Center_Y: 4.33 Å, and Center_Z: 21.26 Å, Size_X: 36.37 Å, Size_Y: 42 Å and Size_Z: 33.75 Å; exhaustiveness: 8; energy range: 3), and (c) fatty acid binding site 1 (Center_X: 41.54 Å, Center_Y: 10.69 Å and Center_Z: 33.55 Å, Size_X: 43.79 Å, Size_Y: 45.38, and Size_Z: 43.53 Å; exhaustiveness: 8; energy range: 3). This study has utilized three independent site-specific docking calculations for HSA with the indomethacin molecule using the Auto Dock Vina program [64]. At the end of the docking calculation, the nine docking poses or docking conformations were generated for each docking calculation and the best docking poses were selected based on the following structural parameters: (a) Δ*G*, (b) *Ki*, and (c) the higher number of docking orientations present at the drug site 1, drug site 2, and fatty acid binding site 1. In order to validate the docking results, the structural superposition analysis was performed between docking solutions with a crystal structure indomethacin-bound HSA structure (PDB ID: 2BXM). Finally, the molecular docking results were analyzed by using programs such as PyMOL (the PyMOL Molecular Graphics System, Version 1.2r3pre, Schrödinger, LLC.), MGLTools (http://mgltools.scripps.edu/; accessed on 10 September 2021), LigPlot [66], and PRODIGY [67] programs, respectively.

Based on the molecular docking results obtained from Auto Dock Vina [64], we found that the first pose of indomethacin of each docking calculation showed more binding affinity towards DS1, DS2, and FA 1 of HSA; therefore, in addition to the molecular docking calculation, we have subsequently performed molecular dynamics (MD) simulations of (a) free HSA, (b) indomethacin bound to DS1 of HSA, (c) indomethacin bound to DS2 of HSA and (d) indomethacin bound to FA1 of HSA with the time period of 100 ns using Gromacs Version 2019 [68].

The physiological behavior of protein or other biomolecular complexes in the presence of water molecules can be mimicked by using MD simulations. The MD simulation begins with a Gromacs formatted file (GRO), topology (TOP), and positional restraint (POSRE) files from the input PDB coordinates of HSA. After that, the cubic box was generated around HSA and HSA–indomethacin complexes. Then, SPCE [69] water molecules were added to the cubic box. Further, counter ions were added to attain the neutral state of the system. If the system is already in a neutral state, this step will be excluded. The number of added water molecules and counter ions for the MD simulation of protein and protein–ligand complexes are given in Table 9.

After this, the steepest descent-based energy minimization was performed for a maximum of 50,000 steps. This step was used followed by two equilibration steps which include NVT (constant number of particles, volume, and temperature; temperature: 300 K; time period: 100 ps; total steps: 50,000) and NPT (constant number of particles, pressure, and temperature; pressure: 1 bar; time period: 100 ps; total steps: 50,000) with the time period of 100 ps, and finally, production MD simulations were carried out with the time period of 100 ns for 50 million steps at a 2 fs time-step. For MD simulation of protein-ligand complexes, initially, the topology (ITP) and Gromacs formatted (GRO) files of indomethacin were developed using the PRODRG external webserver [70]. After the topology building of indomethacin, three independent MD simulations were carried out on protein–ligand complexes with the modifications in the MDP files of energy minimization, NVT, NPT equilibration, and production MD steps. Finally, the comparative MD simulation analyses were performed for (a) free HSA, (b) indomethacin bound to drug site 1 of HSA, (c) indomethacin bound to drug site 2 of HSA, and (d) indomethacin bound to fatty acid binding site 1 of HSA. The following structural analysis were performed with the help of various Gromacs built-in functions such as (a) understand the ligand-induced structural stability (RMSD, RMSF, Rg), understand the ligand-induced binding affinity (H-bonds) and ligand-induced conformation changes of HSA (SASA and secondary structural composition). Apart from the global motion analysis, to understand or capture the biologically significant movements of HSA and indomethacin-bounded HSA structures, we have additionally performed essential dynamics based on principal component analysis.

## 4. Conclusions

HSA contains several binding sites among which indomethacin binds at two sites, one of which, the primary site, is drug binding site 1 located in subdomain IIA, and the other one, designated as the secondary site, is located in subdomain 1B. We have evaluated the quenching and binding constants along with thermodynamic parameters of the binding of indomethacin with HSA corresponding to the primary as well as the secondary site. There was a strong binding between HSA and indomethacin at the primary site while the binding at the secondary site was relatively weaker. Competitive binding site experiments have also shown that indomethacin bound at two different sites. Hydrogen bonding and Van der Waals forces were the dominating forces in the binding at the primary site, whereas, in the case of the low-affinity site, the interaction was mainly favored by hydrophobic forces. The partial unfolding of HSA was taken place when indomethacin was present in larger amounts. Molecular docking and molecular dynamics simulation studies were also in good agreement with the experimental studies.

## Figures and Tables

**Table 1:** Stern–Volmer quenching constants (Ksv1, Ksv2), binding constants (kb1, kb2), and the number of binding sites (n1, n2) obtained from the observed and corrected fluorescence data of HSA–indomethacin interaction at 25 °C by exciting the protein at 295 nm.

	Observed	Corrected
K_SV1_ (mol^−1^)	3.4 × 10^5^ (R^2^ = 0.9952)	3.0 × 10^5^ (R^2^ = 0.9971)
K_SV2_ (mol^−1^)	2.1 × 10^5^ (R^2^ = 0.9861)	1.5 × 10^5^ (R^2^ = 0.9739)
k_b1_ (mol^−1^)	9.3 × 10^5^ (R^2^ = 0.9965)	5.9 × 10^5^ (R^2^ = 0.9966)
k_b2_ (mol^−1^)	5.7 × 10^3^ (R^2^ = 0.9863)	1.9 × 10^3^ (R^2^ = 0.9854)
n_1_	1.08	1.05
n_2_	0.7	0.6

**Table 2:** Stern–Volmer quenching constants (Ksv1, Ksv2), biomolecular quenching constants (kb1, kb2), and binding constants (n1, n2) obtained from the corrected fluorescence data of HSA–indomethacin interaction at 35 °C and 45 °C by exciting the protein at 295 nm.

	35 °C	45 °C
K_SV1_ (mol^−1^)	2.4 × 10^5^ (R^2^ = 0.9952)	2.1 × 10^5^ (R^2^ = 0.9971)
K_SV2_ (mol^−1^)	1.3 × 10^5^ (R^2^ = 0.9861)	1.0 × 10^5^ (R^2^ = 0.9739)
k_b1_ (mol^−1^)	2.9 × 10^5^ (R^2^ = 0.9965)	1.8 × 10^5^ (R^2^ = 0.9966)
k_b2_ (mol^−1^)	1.7 × 10^3^ (R^2^ = 0.9863)	1.4 × 10^3^ (R^2^ = 0.9854)
n_1_	1.00	0.98
n_2_	0.6	0.6

**Table 3:** Stern–Volmer quenching constants and binding constants obtained from the corrected fluorescence data of HSA–indomethacin interaction at 25 °C in the presence of various site markers.

	Warfarin	Ibuprofen	Hemin	Warfarin + Hemin
K_SV1_ (mol^−1^)	1.2 × 10^5^	2.8 × 10^5^	2.1 × 10^5^	1.1 × 10^5^
K_SV2_ (mol^−1^)	-	1.6 × 10^5^	-	6.0 × 10^4^
k_b1_ (mol^−1^)	2.8 × 10^4^	4.7 × 10^5^	1.4 × 10^5^	3.4 × 10^3^
k_b2_ (mol^−1^)	-	1.5 × 10^3^	-	9.8 × 10^2^

**Table 4:** Thermodynamic parameters of the interaction between HSA and indomethacin obtained using the corrected data at the excitation wavelength of 295 nm at the primary and secondary site.

	Primary Site			Secondary Site		
Temp (°C)	25	35	45	25	35	45
∆*G* (KJ mol^−1^)	–32.8	–32.3	–31.9	–18.7	–19.0	–19.2
∆*H* (KJ mol^−1^)	–47.4			–11.6		
∆*S* (J mol^−1^ K^−1^)	–48.9			23.9		

**Table 5:** Estimated binding free energy and the estimated inhibition constant of three sites of human serum albumin with indomethacin.

Name of the Sites	Δ*G* (kcal/mol) (Program: Auto Dock Vina)	*Ki* (μM)	Estimated Binding Affinity (kcal/mol) (Program: Prodigy)
Drug site 1	−10.1	0.04	−6.5
Drug site 2	−7.96	1.46	−5.4
Fatty acid binding site 1	−8.4	0.69	−5.8

**Table 6:** Interacting residue analysis of three sites of HSA with indomethacin.

Name of the Sites	Hydrogen Bonds	Hydrophobic Interactions
Drug site 1 (DS1)	W214 and R218	K195, K199, S202, L203, A210, F211, A215, L238, D451, and L481
Drug site 2 (DS2)	N391	P384, L387, I388, C392, R410, Y411, V433, C437, R445, A449, and R485
Fatty acid binding site 1 (FA1)	None	Leu115, Met123, Phe134, Leu135, Tyr138, Leu139, Ile142, Leu154, Ala158, Tyr161, Phe165, and R186

**Table 7:** Time-averaged structural properties obtained from molecular dynamics simulation of free HSA and indomethacin bound to three sites of HSA.

Protein and Protein–Ligand Complexes	RMSD (Å)	RMSF (Å)	Rg (Å)	HBonds (between HSA & Indomethacin	SASA (nm^2^)	Trace of Covariance Matrix Values (nm^2^)
Free HSA	4.7	1.9	26.5	NA	263.50	278.72
Drug site 1	5.1	1.9	26.1	3	265.12	297.98
Drug site 2	6.2	1.9	26.3	2	270.65	287.46
Fatty acid binding site 1	5.6	1.9	26	1	267.18	273.18

**Table 8:** Secondary structure composition of three sites of human serum albumin with indomethacin (source: gmx dssp).

Protein and Protein–Ligand Complexes	Coil	β-Bridge	Bend	Turn	α-Helix	π-Helix	3_10_-Helix
Free HSA	0.13	0.00	0.00	0.07	0.71	0.01	0.01
Drug site 1	0.14	0.00	0.00	0.08	0.70	0.00	0.01
Drug site 2	0.14	0.00	0.00	0.07	0.70	0.00	0.01
Fatty acid binding site 1	0.14	0.00	0.07	0.07	0.71	0.01	0.01

**Table 9:** Number of added water molecules, counter ions in the molecular dynamics simulation of three sites of human serum albumin with indomethacin (source: Gromacs).

Name of the Sites	Number of Water Molecules Added	Number of Counter Ions Added
Free HSA	43,298	16 NA
Drug site 1	43,272	17 NA
Drug site 2	43,293	17 NA
Fatty acid binding site 1	43,299	17 NA

## Data Availability

Not applicable.

## Supplementary Materials

The following supporting information can be downloaded at: https://www.mdpi.com/article/10.3390/molecules28072979/s1, Table S1. Biomolecular quenching constants (Kq1, Kq2). Figure S1 UV-visible spectra: Figures S2 and S3. RFI at 340 nm: Figures S4–S7. Observed and corrected fluorescence spectra at 35 °C and 45 °. Figures S8–S11. Observed fluorescence spectra of HSA in presence of site markers: figure S12. Root mean square deviation of indomethacin towards three sites of HSA (black—DS1, red—DS2, and green—FA1) Figure S13. Structural superposition of docking solution of indomethacin-bound HSA with the crystal structure of indomethacin-bound HSA (PDB ID: 2BXM) (blue: crystal orientation of indomethacin, red: docking orientation of indomethacin) Figure S14. Two-dimensional protein–ligand interaction of indomethacin towards DS1 of HSA (optimized conformation from 100 ns MD simulation) Figure S15. Two-dimensional protein–ligand interaction of indomethacin towards DS2 of HSA (optimized conformation from 100 ns MD simulation) Figure S16. Two-dimensional protein–ligand interaction of indomethacin towards FA1 of HSA (optimized conformation from 100 ns MD simulation).

## Sample Availability

Samples of the compounds are not available from the authors.
