# Supplementary material for: Detailed Experimental and In Silico Investigation of Indomethacin Binding with Human Serum Albumin Considering Primary and Secondary Binding Sites
Source: Molecules. 2023 Mar 27;28(7):2979. doi: 10.3390/molecules28072979 (PMC10095894; doi:10.3390/molecules28072979)
Supplement: Supplementary file 1 [file molecules-28-02979-s001.zip › molecules-2185779-supplementary.pdf]

Detailed Experimental and in Silico Investigation of Indomethacin Binding with Human Serum Albumin Considering Primary and Secondary Binding Sites

Supplementary Materials

UV–Visible spectra were recorded between the wavelength range of 200 to 400 nm on Perkin-Elmer Lambda 45 Spectrophotometer equipped with autosampler and water-bath with temperature controller. Quartz cuvettes of 1 cm path length were used for the measurements.

Fluorescence measurements were performed on Hitachi spectrofluorometer (Model F 7000) equipped with a PC and programmable temperature controller. Unless stated, the fluorescence spectra were collected at 25 °C with a cell of path length 1 cm. The excitation and emission slits were set at 5 nm. Intrinsic fluorescence was measured by exciting HSA at 280 nm and 295 nm.

The circular dichroism studies of HSA in presence of indomethacin were carried out with JASCO J-815 spectropolarimeter equipped with a Peltier-type temperature controller. The instrument was calibrated with d-10-camphorsulfonic acid. All the CD spectra were collected in a cell of 0.5 mm path-length. The scan speed was 100 nm/min and response time of 1 s for all measurements. Each spectrum was the average of 3 scans.

Equation used for inner filter effect correction:

The observed fluorescence spectra were corrected with following equation:

$$F_{corr} = F_{obs} \times 10^{(A_{exi}+A_{emi})/2} \quad \text{(S1)}$$

where, F_{corr} and F_{obs} are the corrected and observed fluorescence emission intensities, of HSA respectively, A_{exi} and A_{emi} are the absorbances of indomethacin at the excitation and emission wavelengths, respectively.

Equation used to calculate thermodynamic parameters:

van 't Hoff equation is given as:

$$\ln K_b = \frac{-\Delta H}{RT} + \frac{\Delta S}{R} \tag{S2}$$

$$\Delta G = \Delta H - T\Delta S \tag{S3}$$

where ΔH is enthalpy change, ΔS is entropy change and ΔG is free energy change. R is gas constant and T is temperature in K.

Table S1. Biomolecular quenching constants (Kq1, Kq2) of HSA-indomethacin interaction by exciting the protein at 295 nm.

	Temperature		
	25°C	35°C	45°C
K_{q1} (mol^{-1})	5.1×10^{13}	4.1×10^{13}	3.6×10^{13}
K_{q2} (mol^{-1})	2.5×10^{13}	2.2×10^{13}	1.7×10^{13}

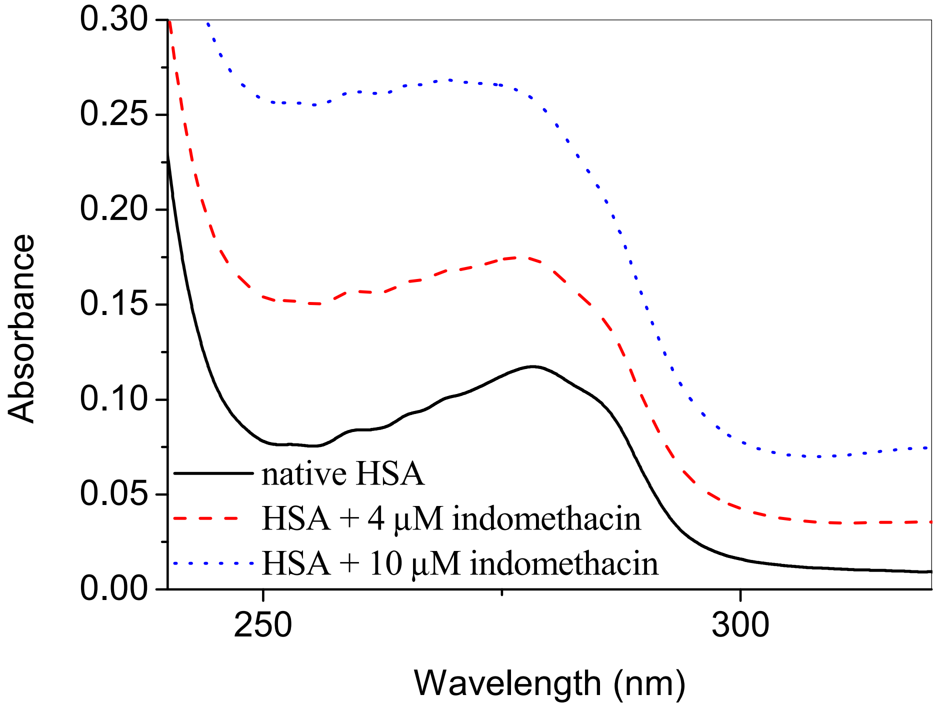

Figure S1. UV-visible spectra of HSA in absence and presence of indomethacin at 25 °C.

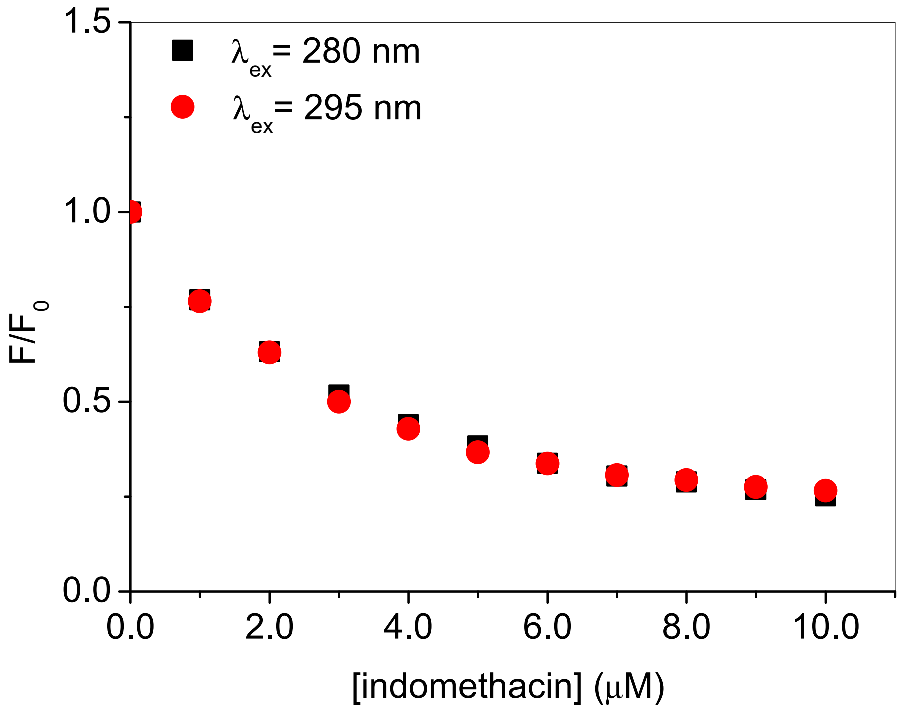

Figure S2. RFI of HSA fluorescence at 340 nm obtained from the observed data at 25 °C at two excitation wavelengths.

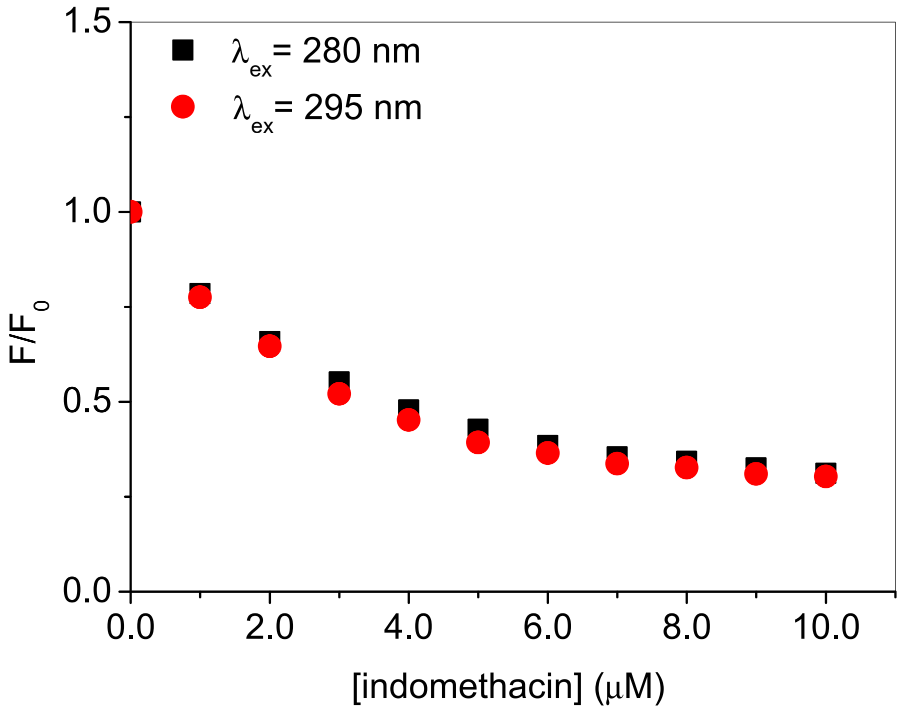

Figure S3. RFI of HSA fluorescence at 340 nm obtained from the corrected data at 25 °C at two excitation wavelengths.

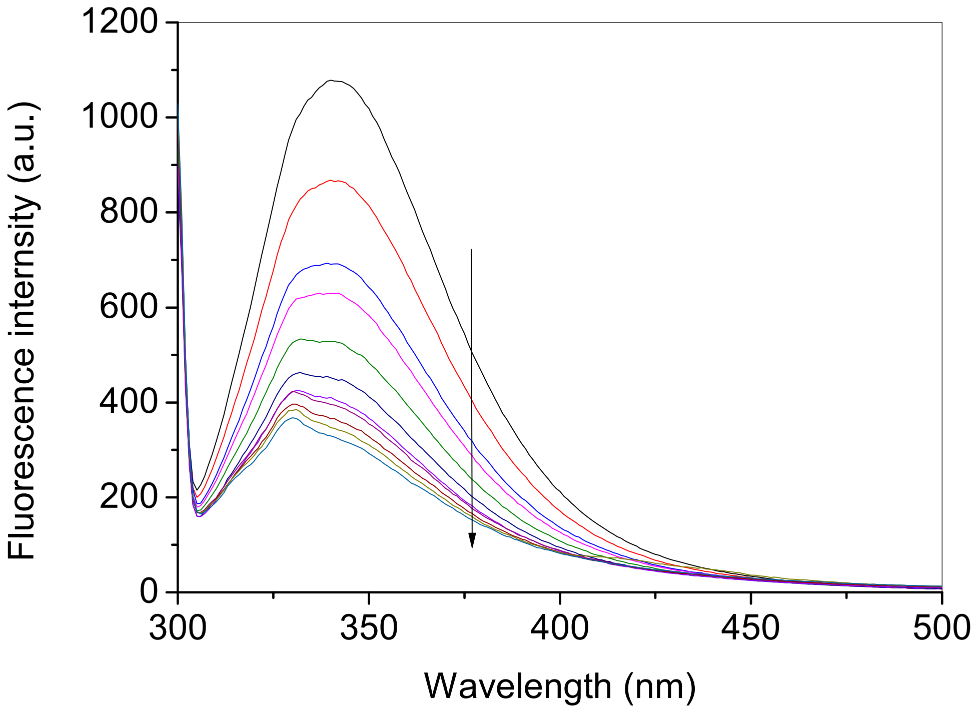

Figure S4. Observed fluorescence spectra of HSA in absence and presence of indomethacin at λ_{ex} of 295 nm at 35 °C. [HSA] = 3 µM and [indomethacin] = 0. 1.0, 2.0, 3.0, 4.0, 5.0, 6.0, 7.0, 8.0, 9.0 and 10.0 µM.

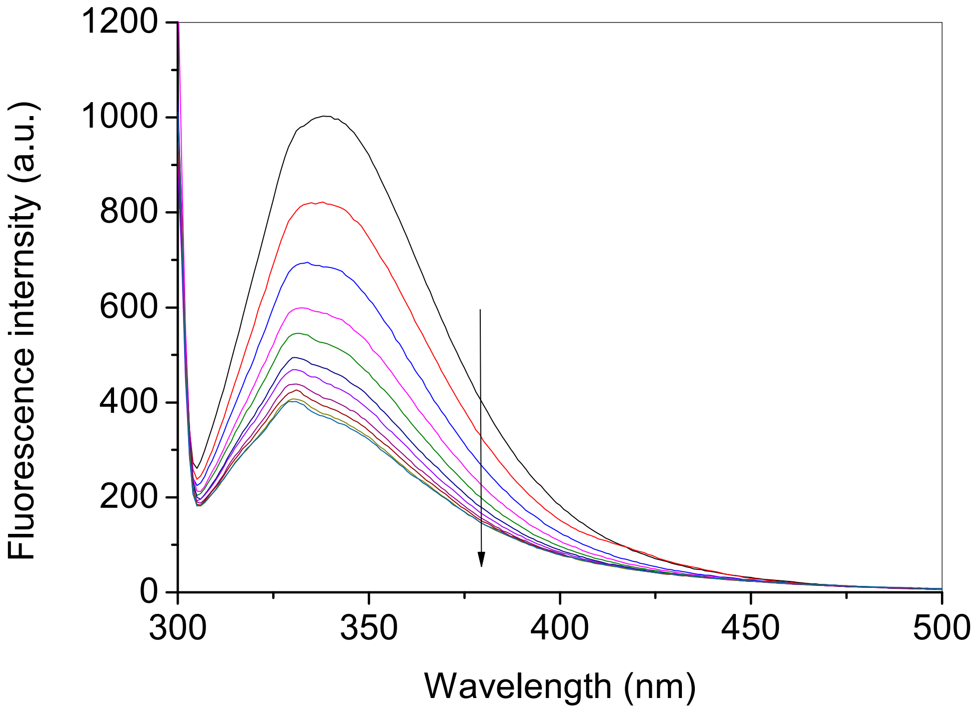

Figure S5. Observed fluorescence spectra of HSA in absence and presence of indomethacin at λ_{ex} of 295 nm at 45 °C. [HSA] = 3 μM and [indomethacin] = 0. 1.0, 2.0, 3.0, 4.0, 5.0, 6.0, 7.0, 8.0, 9.0 and 10.0 μM.

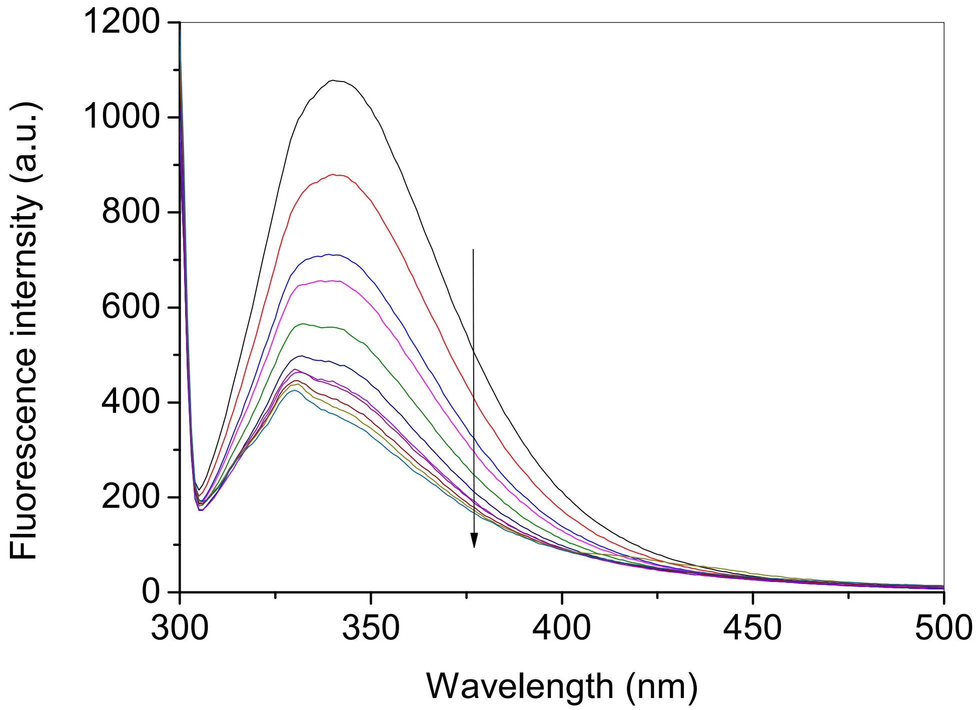

Figure S6. Corrected fluorescence spectra of HSA in absence and presence of indomethacin at λ_{ex} of 295 nm at 35 °C. [HSA] = 3 μM and [indomethacin] = 0. 1.0, 2.0, 3.0, 4.0, 5.0, 6.0, 7.0, 8.0, 9.0 and 10.0 μM.

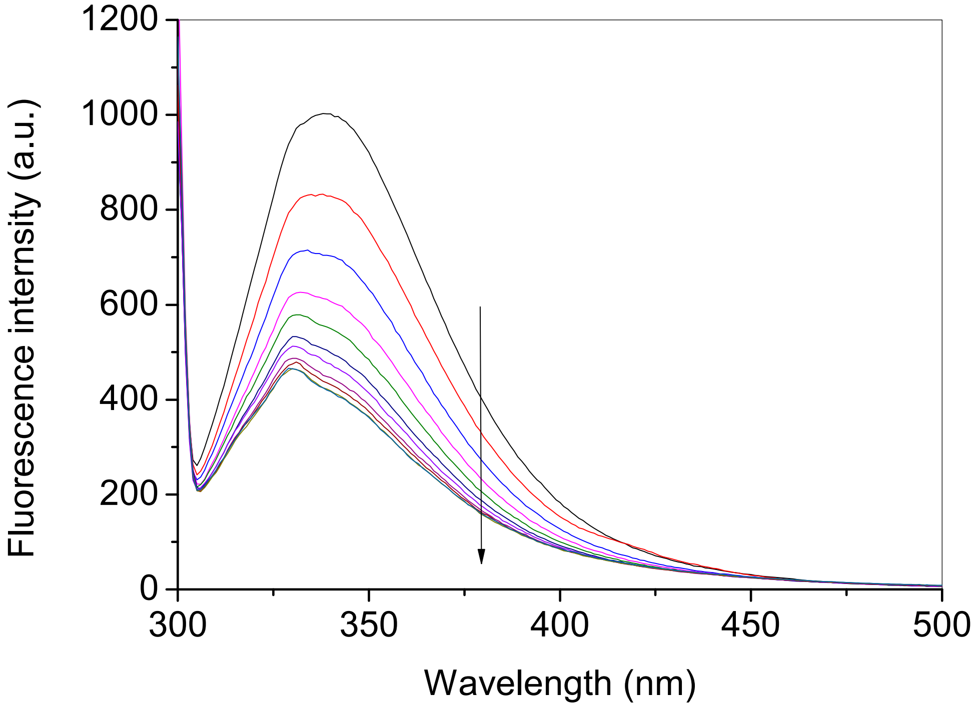

Figure S7. Corrected fluorescence spectra of HSA in absence and presence of indomethacin at λ_{ex} of 295 nm at 45 °C. [HSA] = 3 μM and [indomethacin] = 0. 1.0, 2.0, 3.0, 4.0, 5.0, 6.0, 7.0, 8.0, 9.0 and 10.0 μM.

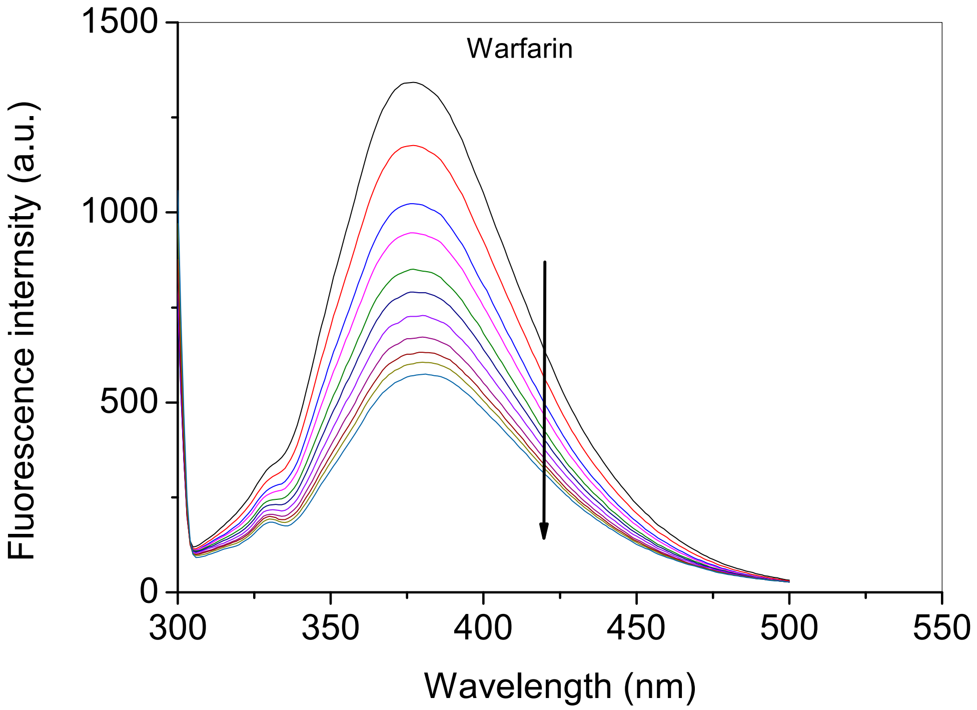

Figure S8. Observed fluorescence spectra of HSA-warfarin system in absence and presence of indomethacin at λ_{ex} of 295 nm at 25 °C. [HSA] = 3 μM and [indomethacin] = 0. 1.0, 2.0, 3.0, 4.0, 5.0, 6.0, 7.0, 8.0, 9.0 and 10.0 μM.

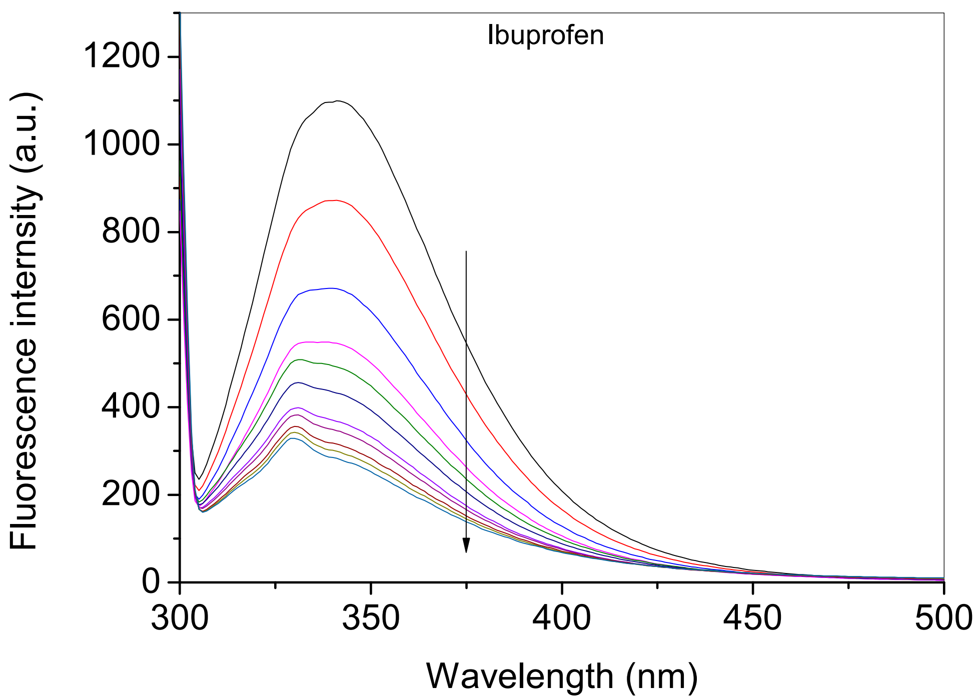

Figure S9. Observed fluorescence spectra of HSA-ibuprofen system in absence and presence of indomethacin at λ_{ex} of 295 nm at 25 °C. [HSA] = 3 μM and [indomethacin] = 0. 1.0, 2.0, 3.0, 4.0, 5.0, 6.0, 7.0, 8.0, 9.0 and 10.0 μM.

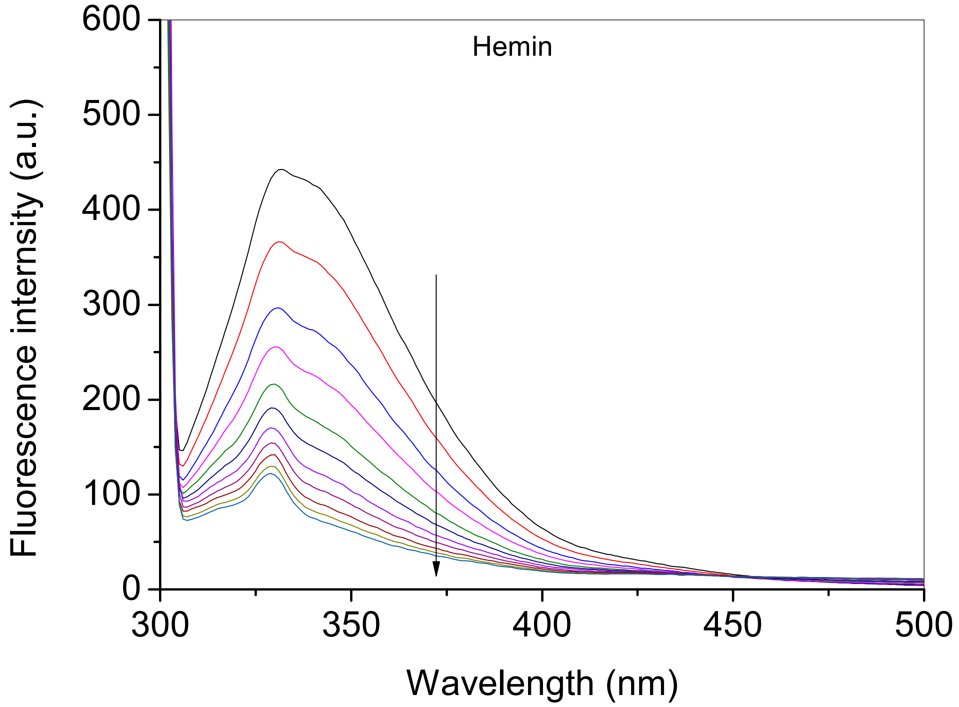

Figure S10. Observed fluorescence spectra of HSA-hemin system in absence and presence of indomethacin at λ_{ex} of 295 nm at 25 °C. [HSA] = 3 μM and [indomethacin] 0. 1.0, 2.0, 3.0, 4.0, 5.0, 6.0, 7.0, 8.0, 9.0 and 10.0 μM.

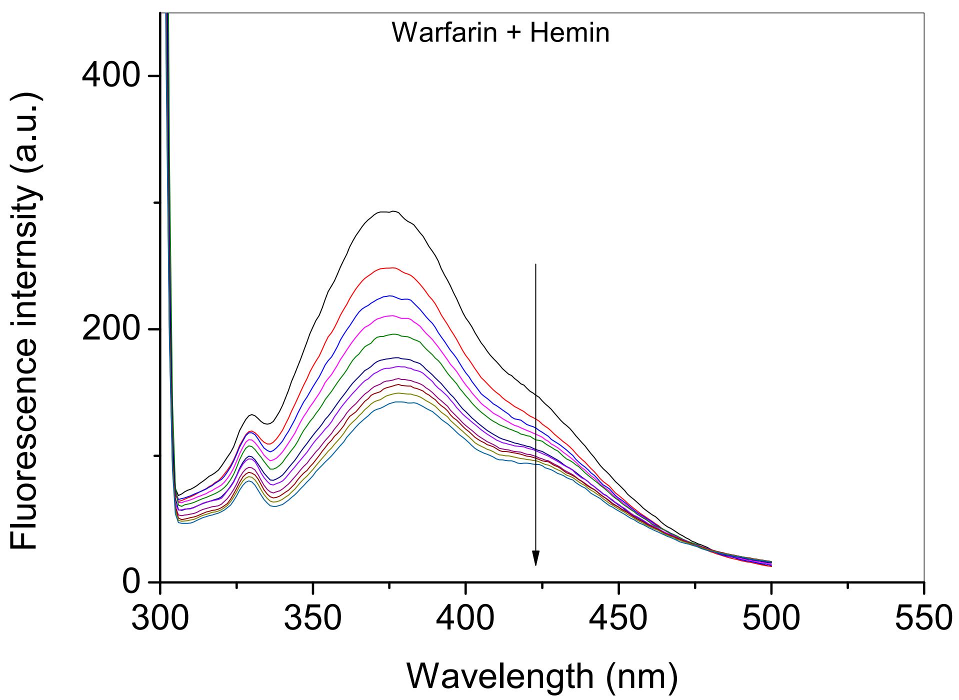

Figure S11. Observed fluorescence spectra of HSA-warfarin-hemin system in absence and presence of indomethacin at λ_{ex} of 295 nm at 25 °C. [HSA] = 3 μM and [indomethacin] = 0. 1.0, 2.0, 3.0, 4.0, 5.0, 6.0, 7.0, 8.0, 9.0 and 10.0 μM.

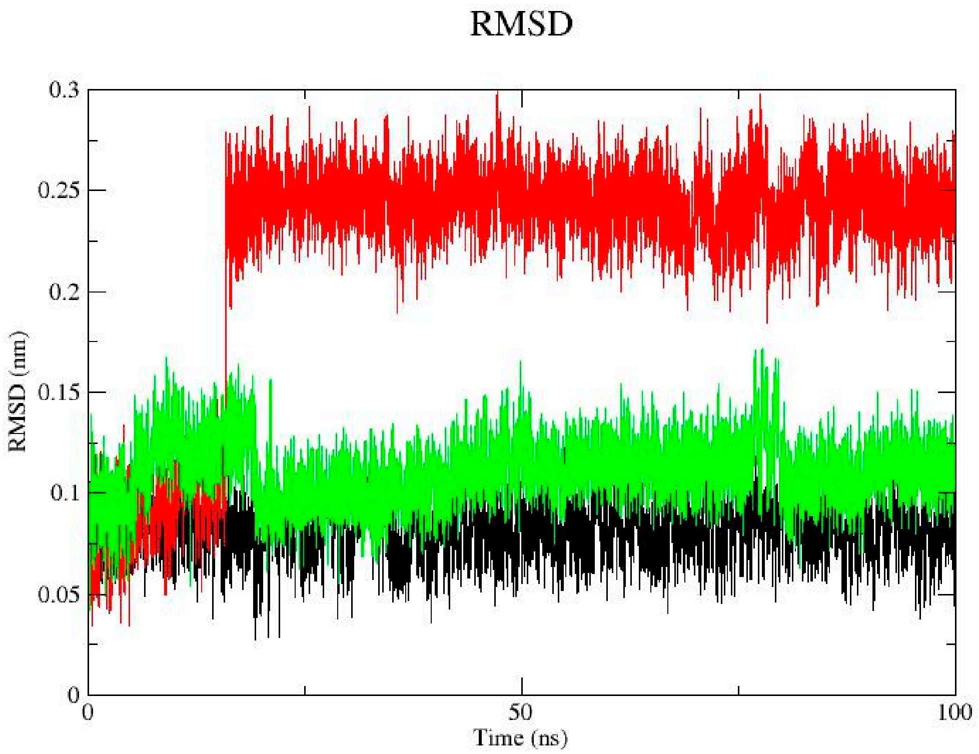

Figure S12. Root Mean Square Deviation of indomethacin towards three sites of HSA (Black-DS1, Red-DS2 and Green-FA1)

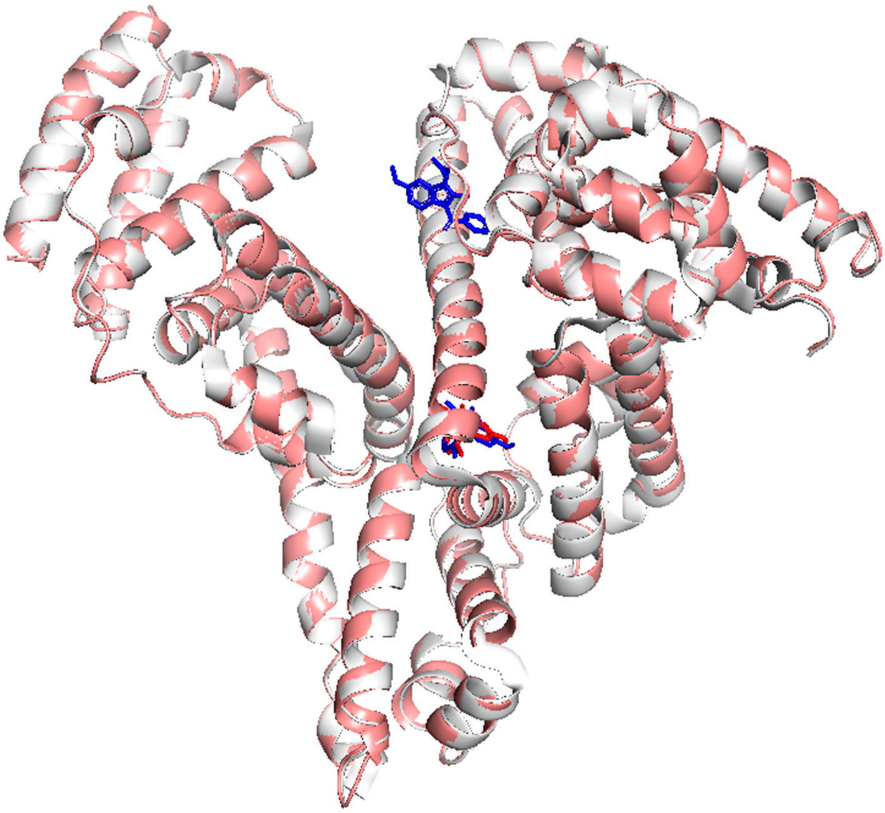

Figure S13. Structural superposition of docking solution of indomethacin bound HSA with crystal structure of indomethacin bound HSA (PDB ID: 2BXM) (Blue: Crystal orientation of indomethacin, Red: Docking orientation of indomethacin)

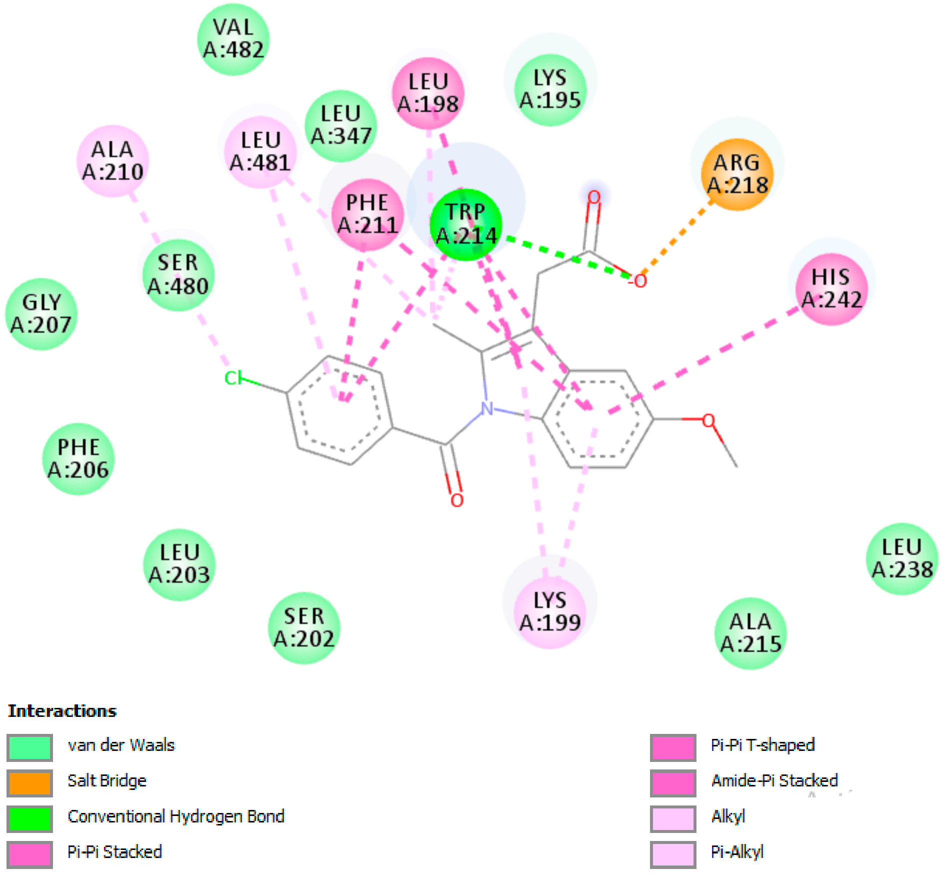

Figure S14. Two-dimensional protein-ligand interaction of indomethacin towards DS1 of HSA (optimized conformation from 100ns MD simulation)

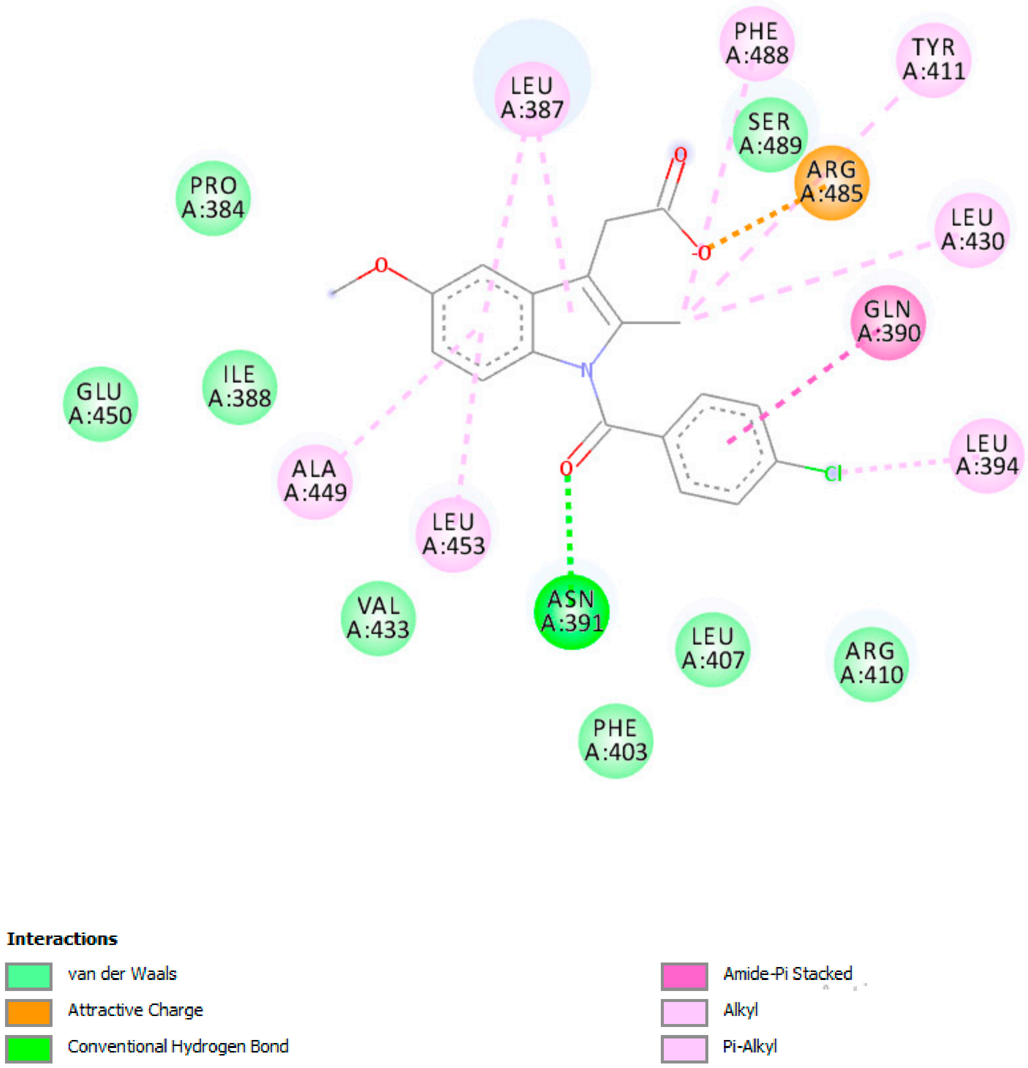

Figure S15. Two-dimensional protein-ligand interaction of indomethacin towards DS2 of HSA (optimized conformation from 100ns MD simulation)

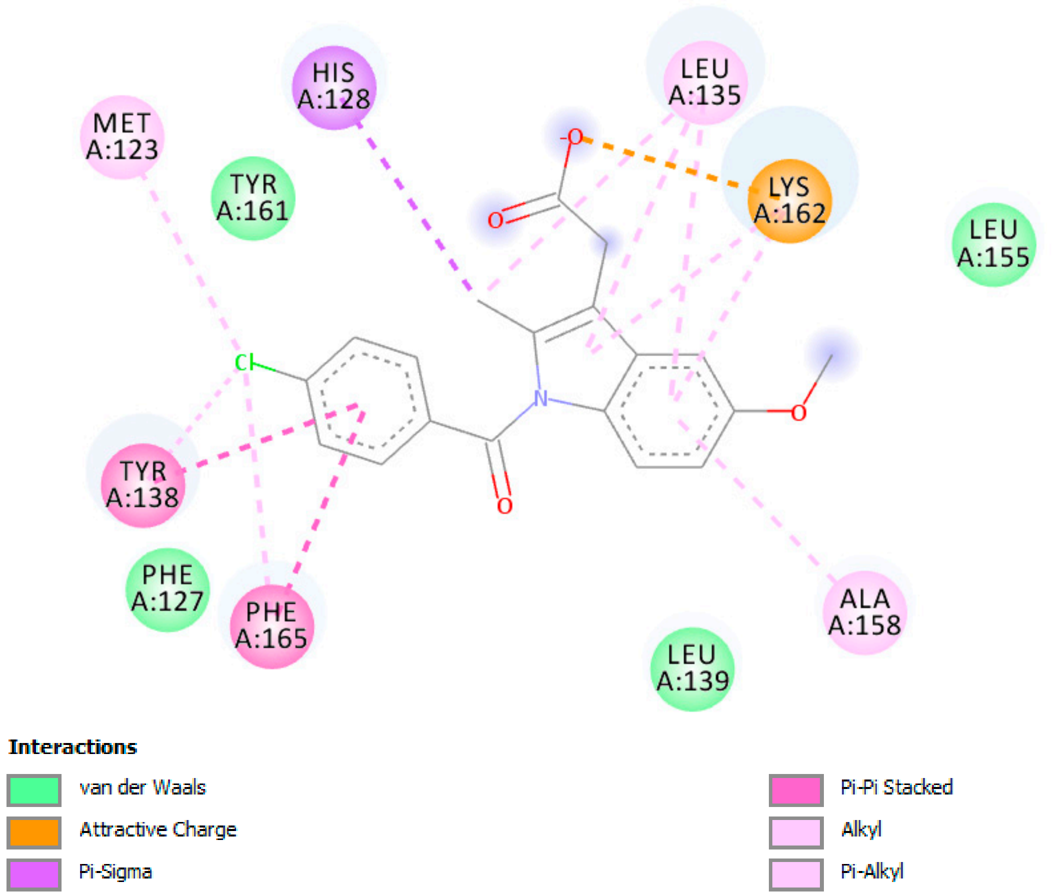

Figure S16. Two-dimensional protein-ligand interaction of indomethacin towards FA1 of HSA (optimized conformation from 100ns MD simulation)
